# Integrating Wi-Fi, Li-Fi, and BPL Technologies for a Secure Indoor Communication System

**DOI:** 10.3390/s24248105

**Published:** 2024-12-19

**Authors:** Mostafa Eltokhy, Ali M. El-Rifaie, Heba Allah Gamal, Ayman Haggag, Hisham Ali, Ahmed A. F. Youssef, Ashraf Aboshosha

**Affiliations:** 1 Electronics Technology Department, Faculty of Technology and Education, Helwan University, Cairo 11792, Egypt; heba.gamal@techedu.helwan.edu.eg (H.A.G.); haggag@techedu.helwan.edu.eg (A.H.); hisham.ali@techedu.helwan.edu.eg (H.A.); 2 College of Engineering and Technology, American University of the Middle East, Egaila 54200, Kuwait; ahmed.youssef@aum.edu.kw; 3 Radiation Engineering Department, National Center for Radiation Research and Technology (NCRRT), Egyptian Atomic Energy Authority (EAEA), Cairo 11371, Egypt; ashraf.aboshosha@eaea.org.eg

**Keywords:** broadband over power lines (BPL), light fidelity (Li-Fi), wireless fidelity (Wi-Fi), smart grid, sustainable communication technologies

## Abstract

In today’s digital age, there is an increasing demand for integrated wireless and wired technologies; however, there is a difficulty in achieving secure and reliable communications within buildings and facilities. This paper presents a proposal for maintaining the infrastructure while expanding it to implement communication technologies with high transmission and reception speeds and high levels of data confidentiality to enhance the operational efficiency of organizations. Three main technologies have emerged as promising solutions for this purpose: Wi-Fi, Li-Fi, and BPL. Despite the advantages that each technology offers, some drawbacks appear in these technologies that affect data transmission. Wi-Fi, Li-Fi, and BPL can be combined to achieve maximum security and reduce noise and interference via the ESP8266 module. This combination could be an important step toward achieving an integrated and secure indoor communication system. The paper provides a comprehensive overview of the three techniques and how they are applied in practice. In addition, OSE, ABPF, and ASBF filters are used to detect and eliminate interference and attack secure internal networks.

## 1. Introduction

Due to the increasing number of users and connected devices, it has become difficult to manage networks and ensure the effective provision of services. Several basic problems have also emerged in communications systems, such as signal interference, which affects the quality of communication and data speed; the increase in hacking and cyber-attacks that threaten the integrity of data; the high costs of installing and maintaining new infrastructure; the challenges of network expansion and compliance with laws and regulations related to communications, which prevents some companies from updating their systems; and environmental problems such as electromagnetic interference, storms, etc., which greatly affect network performance.

Many modern technologies have emerged and are now used in advanced networks, smart cities, basic electrical networks, and other applications, including the smart electrical grid, which works to transform traditional electricity distribution networks into advanced and integrated systems. It benefits from modern technology, enhances energy sustainability, provides electricity, and enhances efficiency and flexibility in the grid and its effective management of the electric power system using digital and communications technology. This is performed by using modern technologies such as wireless communications technologies, cellular networks, optical fiber technologies, and other communications technologies that enable real-time monitoring and control of electrical power generation, transmission, distribution, and consumption, and enabling continuous interaction between consumers and the electricity network [1].

The proper application of smart grids and the use of modern technology will increase the reliability, efficiency, safety, and sustainability of the electric power distribution system and all that it includes in terms of power generation, consumption, and storage from multiple sources; demand response programs; automatic fault detection in the network; data analysis and rapid response to changes; and implement automatic processes to redirect power supply, avoid power outages, and provide information to consumers to enhance their participation [2]. One of the technologies that smart grids and advanced networks rely on now to transmit data is optical fiber. It increases the speed of the network, which is in the range of a gigabit until it reaches a terabit for data transfer, and works to distribute power across the network, in addition to the high bandwidth and degree of high security, increased network stability and reliability, network scalability, reduced attenuation, and electromagnetic interference (EMI) [3].

There are also many technologies that can be used across energy transmission and distribution networks, such as wireless communications technologies, which are wireless local networks (Wi-Fi), wireless sensor networks (WSNs), internet networks (DSL), portable personal networks (PANs), and wide local area networks (WLANs) to transfer data [4]. Cellular generation networks such as fourth generation (4G) and fifth generation (5G) can also be used to transfer data at high speeds and wide coverage, which supports communication between devices connected to the smart network and smart control centers in different regions and wired communications networks can be used, such as fixed telephone lines, copper cables, and Ethernet networks. Data transmission techniques via power lines (PLC) have also been recently added to transmit data in the smart grid and, as a result, new technologies such as Li-Fi and BPL can be added to improve network efficiency and provide more comprehensive and high-quality communication solutions, not only with smart networks but with all applications.

To suit all needs, these technologies enhance compatibility between devices, increase network flexibility, and achieve a balance between the performance and network cost. They can operate as independent technologies, be used as backup technologies for each other in the event of a failure in one of the technologies, or be integrated with each other. Examples of the potential for technology integration include the use of fiber-optic cables to connect key areas in the smart grid, while cellular communication technologies such as 4G or 5G can be used to connect sensors in remote areas or environments where fiber-optic cables are difficult to extend [4].

Despite the advantages of Li-Fi and BPL technologies, they differ from each other in terms of compatibility, technical integration, different standards, performance efficiency, implementation and operation costs, transmission speed, coverage, and available infrastructure, which represents a challenge when integrating them together. In this paper, the proposed technologies (BPL, Li-Fi, and Wi-Fi) will be used to work as separate technologies or to integrate them, to make the most of these different technologies to obtain a secure and reliable indoor communication system with high communication quality.

The paper is organized as follows. In Section 2, an introduction to Li-Fi technology is given, while in Section 3, an introduction to the BPL technique and a comparison between the three proposed techniques is presented. Section 4 shows how to apply simplified electronic circuits to achieve integration between the proposed technologies across core energy networks. Section 5 explains how to protect the network by detecting and stopping threats, especially in security and military networks. Section 6 discusses applications that can be implemented to achieve this integration. Section 7 presents the conclusions.

## 2. Li-Fi Technology

Li-Fi technology is short for light fidelity, which is a meaning close to the term “dependence on light”. It is one of the wireless communication systems based on visible light known as “visible light communication” (VLC), which was first shown by the scientist Alexander Graham Bell in 1880 through the optical telephone that he invented, which transmitted voice data at more than 200 m using sunlight [5]. Harald Haas introduced Li-Fi technology, which showed that light can be an effective medium for high-speed data transmission, by unifying the PHY and MAC layers to handle the optical signals and the actual interaction between devices [6], this helped in coordinating data transfer between devices, reduce interference from ambient light and enhance security [7].

Li-Fi depends on visible light to transmit data. Visible light falls within the electromagnetic spectrum between infrared (IR) and ultraviolet (UV) rays. Its frequency is between 430 and 790 THz and corresponds to wavelengths of about 750 nm to 380 nm. This is what causes it to have a large capacity that can be used for various communications systems, 10,000 times greater than the range of radio waves and micro waves [8].

Visible light does not affect human health unlike radio waves. Visible light can also be obtained through LED lamps, which are now available everywhere because they are environmentally friendly, have low cost, and have low energy consumption. Moreover, their lighting is intermittent at very high rates, and our vision does not perceive this intermittence. This is useful in obtaining binary logic (0 or 1), so the high-speed changes in light intensity of LED lamps allow the transfer of information in the form of light signals as a wireless channel, and thus the possibility of using them for lighting and transferring data simultaneously [9].

Li-Fi technology is free from electromagnetic interference, so it is best considered for devices that are sensitive to electromagnetic interference used in industrial, healthcare, aviation, military applications, etc. [8]. Due to the increasing demand for the use of limited electromagnetic radio frequencies and the demand for network expansion, especially after the emergence of the Internet of Things, it is considered a better option than radio technologies, as it does not require infrastructure because lighting is already present, and this may be the reason for the development of 5G/6G generations [10,11,12]. This makes it easy to implement with low cost and low power consumption. However, due to the difficulty of optical signal penetration and its non-passage through walls, and the very low access time because the transmission distances are short, Li-Fi can be used as an alternative to Wi-Fi or as a complement to it, because Li-Fi access points are much better than traditional Wi-Fi technology [7].

Li-Fi technology works over short distances [8]. The range of the signal is limited inside the place only, depending on the LOS transmission path. Although this is a flaw in this technology, it is useful in making communication secure [12]. Interference may occur between the signal and other light sources with the signal, such as sunlight interfering with the signal.

**Figure 1:**
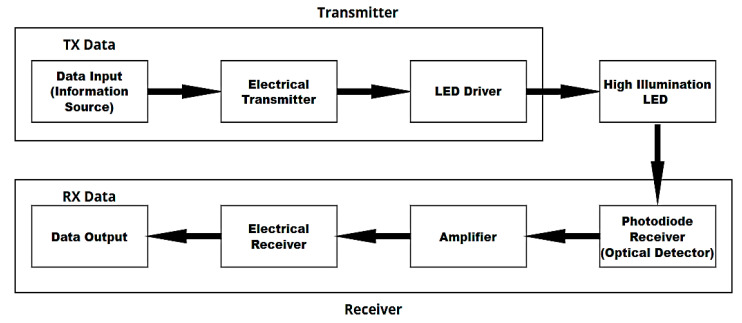
Basic block diagram of a Li-Fi system.

In Figure 1, the schematic diagram shows the basic components of Li-Fi technology, which must be present to achieve a Li-Fi communication system. It consists of three basic sections [4,9,10] as follows:

First: the transmitter side, in which the data are converted into electrical signals, then converted into binary data through the ADC converter, then converted to the LED Driver, which processes and modifies the signal.

Second: The communication channel, which is represented by the visible light coming from an LED or LED lamp. They are used for lighting in addition to being used as a data carrier at rates of up to 100 gigabits per second. It works to convert the signal into a light signal. As a result of its on and off pattern while maintaining its high brightness due to the presence of the modulation, the light signal then passes through a communication channel.

Third: The receiving side, where the photodetector receives the optical signal and works to convert the signal to an electrical signal. Then, the modification and decoding are removed, and the signal is converted back into an analogue signal through DAC. The signal is then amplified and filtered from any frequencies other than desirable to recover the data sent.

The data transfer speed in Li-Fi is much higher than other wireless communication technologies such as Wi-Fi. This speed is related to many factors, including the quality and efficiency of the LED lamps used and the techniques used for modifying and controlling optical signals. Any delay in the process of converting the signal from electrical to optical will affect the speed of data transfer, as shown in Table 1, which presents the comparison between Li-Fi technology, Wi-Fi technology, and BPL technology [13].

## 3. BPL Technology

**Figure 2:**
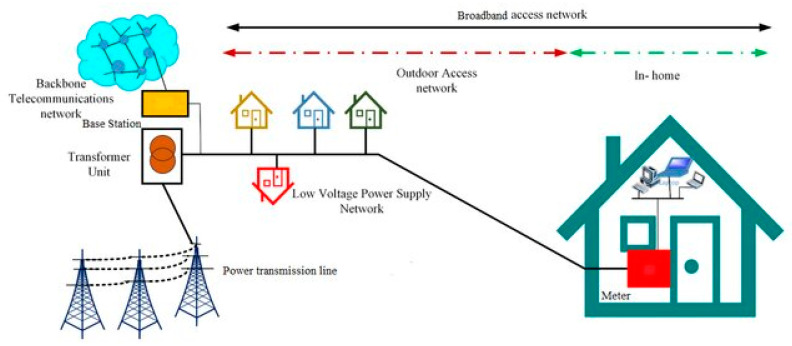
Architecture of broadband over power lines.

Broadband over power lines (BPL), is a power line communication (PLC) technology that works to transfer data through medium- and low-power transmission lines (electricity distribution infrastructure), by combining the frequencies of the signal to be sent, which are often high-frequency radio frequencies (RF), on the same power transmission line that carries alternating current (220 V, 50 Hz). It also does not require the rearranging of the power line cables or making any changes to them [14]. In addition, it will not cause interference between the two signals, and the data packets that are sent or received are not subject to loss or noise due to electrical current. Figure 2 shows the architecture of BPL technology [14].

BPL has become widely used within small networks such as nuclear plants, military institutions, and financial institutions, and in industrial and commercial applications such as smart grids, and others. Although the use of BPL technology is not as common as other communication technologies such as fiber optics, DSL technology, or wireless communications, it is characterized by ease of installation, implementation, expansion, and low cost of infrastructure presence [4]. It is used as an alternative to providing internet service in areas where access to internet services is not available, such as remote and rural areas or military institutions and nuclear plants, which are characterized by a high degree of security and speed with speeds reaching Gbps [15].

The reason why it is not commonly used is due to the laws and regulations of each country, in addition to technical challenges such as the interference of data signals with other wireless communication systems that use similar frequencies, and thus the appearance of noise, a decrease in signal quality, and the appearance of attenuation resulting from long or complex power lines [16]. 

The mechanism of operation of BPL depends on converting the data to be sent into electrical signals transmitted through the electrical network, and special equipment called BPL modems are used to convert signals between the electrical network and the means of displaying the data used [17,18]. Once the computer or any data display device is connected to the BPL modem, which is connected to any socket in the building, the desired data can be received at high speeds and high frequencies through the power line, which works to transfer and distribute energy with the ability to transfer and exchange data at the same time. This means that it depends on connecting the transmitting modem (transmission circuit) to one of the ports of the building’s main electrical network, and it uses the existing electrical network to transmit high-frequency signals as a communication channel that works to transfer data to the receiving modem (receiving circuit) located on the same electrical network, with the possibility of using communication technologies that provide a display data. 

BPL can provide high-speed internet and transmit a high-frequency signal in the range of 1.6: 30 MHz with low-level power and transmit it via a power line with low-frequency and high-power levels (50–60 Hz, 220 V) [18].

## 4. System Design

This paper proposes the use of Wi-Fi, Li-Fi, and BPL technologies to work as independent or complementary technologies to each other. The goal of using these technologies together is to provide a wired and wireless network that covers large areas, is low cost, has low power consumption due to its reliance on infrastructure, and can be easily accessed by various devices, supports high data transfer speeds, making it suitable for applications such as live streaming, and high levels of network security with access control through multiple authentication strategies and security protocols. Every technology has its challenges, such as Li-Fi, which relies on visible light, so the signal remains confined to closed spaces only, and may be affected by sunlight during the day; therefore, it is possible to integrate it with other technologies such as Wi-Fi. Also, BPL, when used without securing the network, may increase the chances of spying and hacking, but when integrated with Li-Fi, it makes the network secure against any threats. By implementing the network using the proposed integration techniques, this creates a more secure communications environment suitable for use in sensitive, security, and military environments. The circuits are designed with low-cost, easy-to-use components and rely on their own infrastructure and network system architecture.

A. Li-Fi system design

**Figure 3:**
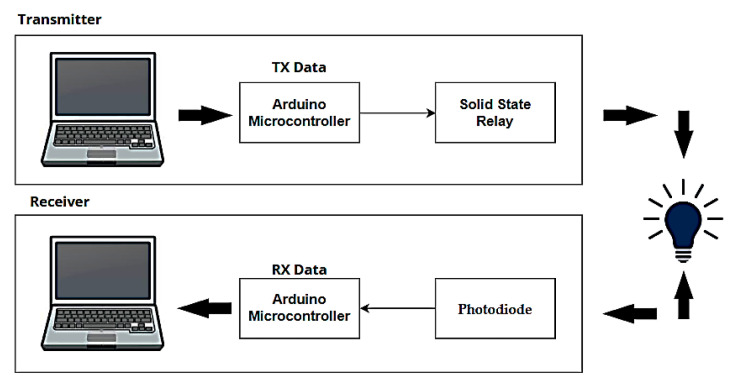
Proposed block diagram of a Li-Fi system.

A Li-Fi system based on the use of LEDs to transmit data via the visible light spectrum has been proposed by designing two circuits, one for transmitting and the other for receiving Li-Fi signals via an Arduino Uno controller capable of transmitting digital data by connecting it to a computer via a USB cable, through which text, image, and video signals are successfully sent and received. Figure 3 presents a block diagram designed to implement a Li-Fi system, showing a transmitter and receiver units based on visible light. It is like the block diagram in Figure 1, but with the addition of an Arduino controller, using a solid-state relay to achieve on–off keying (OOK) modulation, to adjust the light intensity of an LED.

During implementation, it was found that an LED can be used as an alternative to a photoreceptor when light is shining on it. It can generate simple electricity of up to 2 V, depending on the type of LED used. The reason for this is that the internal structure of the solar cell is like that of an LED. But it is not preferable to use it because when sending data, it depends on the presence of logic 1 or 0, and therefore the controller will always read 0 because of the voltage being less than 5 V. This can be solved by using an amplification circuit that amplifies the received signal, but to be compatible with all systems and to reduce the cost, a photodiode was used as a photodetector to receive the data.

**Figure 4:**
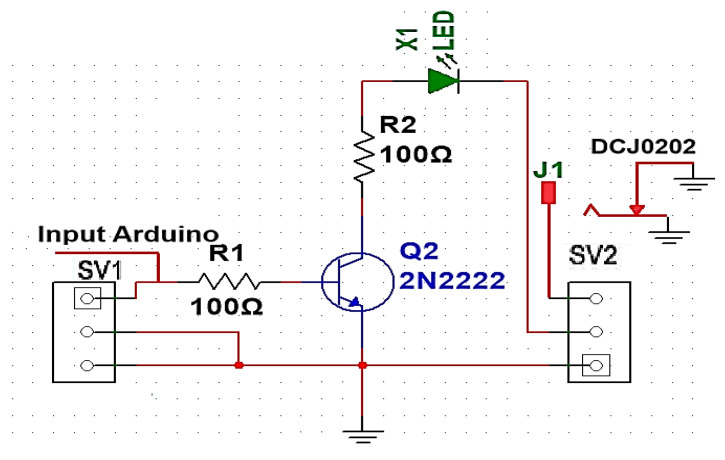
Proposed Li-Fi transmitter circuit.

Figure 4 shows the proposed Li-Fi transmitter circuit. Solid-state relay (SSR) was used with Li-Fi technology. It uses OOK modulation, which in turn modulates the intensity of the LED lamp (i.e., controls the illumination of the LED lamp), which acts as data transfer points by operating at high speed to create light pulses that carry data. Through SSR, LED lamps can be turned on and off at high speed and with great accuracy for high-speed data transmission by converting the optical signal into an electrical signal to control the operation of the lamp [19]. This is very similar to sending data via Morse code to send intermittent light pulses at very high speeds. 

The lamp is on all the time, but when data is sent, it turns on again at a very high speed and for a specific period. The longer the word, the longer the lamp is turned off. Each word has a specific time to be sent. For example, sending a Wi-Fi word that turns the lamp on and off takes 100 ms, a Li-Fi word takes 500 ms, while BPL takes 700 ms, meaning that the lighting changes because of the sent data. The speed and format of the sent data can also be adjusted by programming the Arduino and, thus, this can be exploited in some applications, such as alerting people with special needs that there is data to receive.

**Figure 5:**
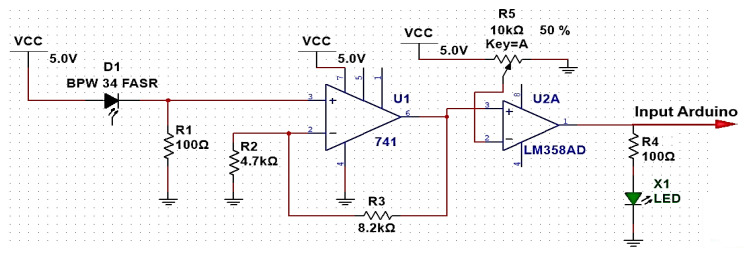
Proposed Li-Fi receiver circuit.

Figure 5 shows the proposed Li-Fi receiver circuit, where the data sent is received through the photodiode, which converts the optical signal into an electrical signal. This signal has a low value, so this signal is amplified through an amplification circuit and then to a comparator circuit in order to obtain the appropriate data signal that is stable, even in the case of a long distance between the transmitter and receiver circuits, and then it is converted into binary data to be connected via the Arduino controller connected to the receiving computer to display the data.

**Figure 6:**
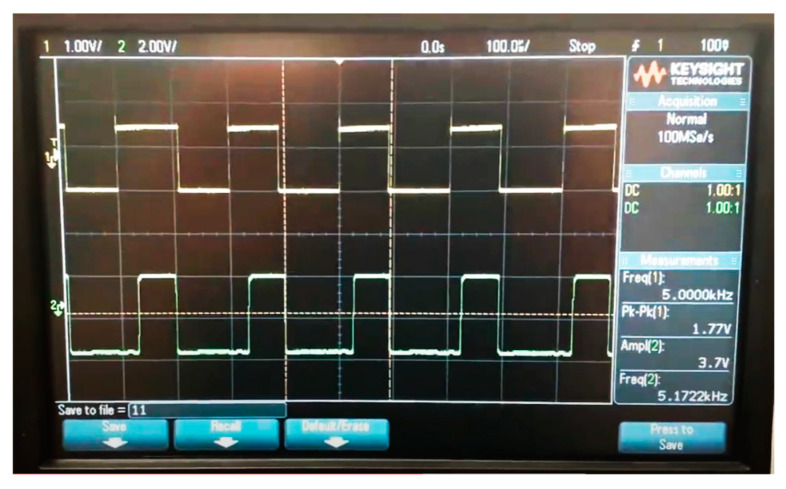
Signal transfer from the transmitter to the receiver.

Figure 6 shows how well the transmitted and received signals propagated from the Arduino match the proposed Li-Fi system when sending a text signal. The first signal in yellow is the transmitted signal while the second signal in green is the received signal. Some differences may occur between the two signals due to noise and capacitive effects that affect the received signal. Li-Fi systems generally use optical signals for transmission and electrical signals for reception, which leads to obvious differences between the transmitted and received signals when handling data.

**Figure 7:**
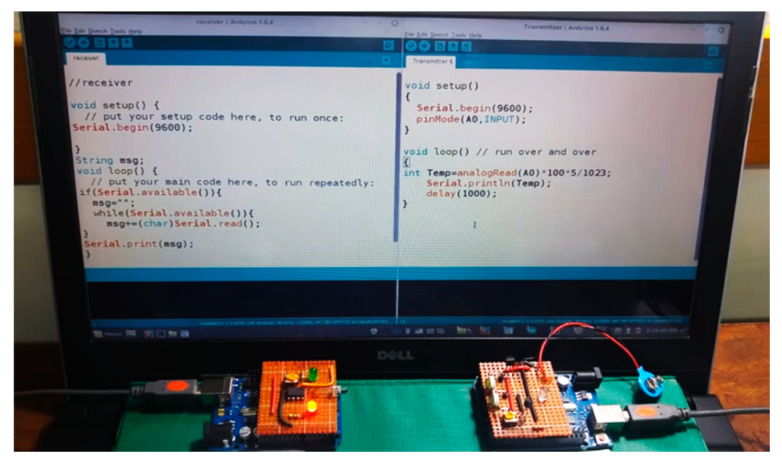
The practical implementation of a LI-Fi system.

Figure 7 shows the practical implementation of the proposed Li-Fi system. To implement a Li-Fi network, the existing LED lighting network is connected to the building, so that users can receive data via a single LED or move from one LED to another without affecting their connectivity. For greater security, reliability, and data protection from hacking, encryption through Python was used. In this case, the message will be sent first to be encrypted by the sender and then to be decrypted by the receiver.

B. Integration between Li-Fi and Wi-Fi

As a result of the challenges facing Li-Fi technology [12], which were previously mentioned in Section 2 and Table 1, Li-Fi requires a direct line of sight between the source (such as the LED) and the receiver (such as the photodiode), which may be a challenge in some environmental conditions. Although Li-Fi provides great security due to the lack of light spreading outside closed spaces, it is affected by other light sources such as sunlight, which may lead to significant data loss during transmission. Therefore, Wi-Fi technology can be used as complementary technology, especially during daylight hours [7].

**Figure 8:**
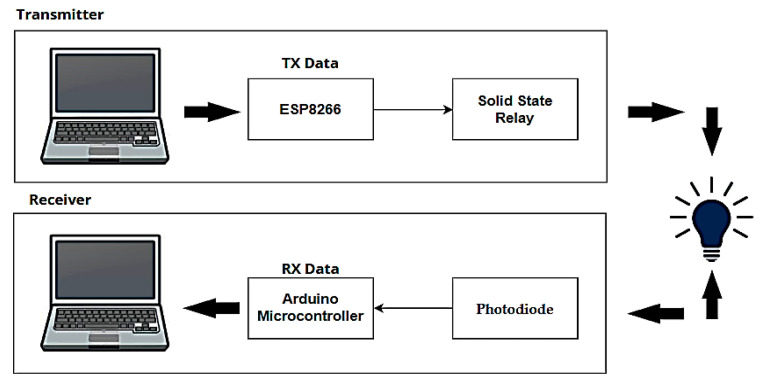
Proposed block diagram of the integration between Li-Fi technology and Wi-Fi technology.

Challenges may arise when performing this integration, such as different protocols and standards, compatibility with devices, and interference between Wi-Fi wireless signals and Li-Fi optical signals, which affects the quality of communication in shared environments. However, the implementation of this proposed integration is characterized by the possibility of network management and connection stability, by linking data to light with the possibility of displaying it on a webpage for easy access. The network can be designed so that Li-Fi devices are placed in locations that ensure a direct line of sight with minimal interference with Wi-Fi. Figure 8 shows the proposed schematic diagram for implementing the integration of Li-Fi and Wi-Fi technologies.

**Figure 9:**
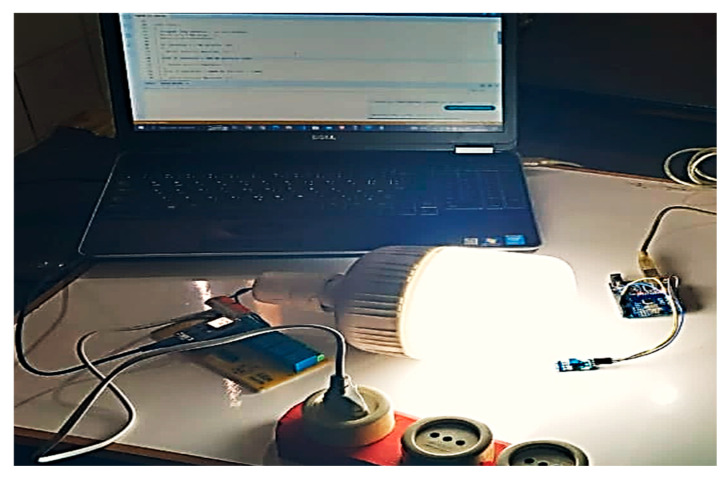
The practical implementation of integration between Li-Fi and Wi-Fi technology.

An ESP8266 chip was used, which is a small, low-cost, easy-to-use microcontroller model. It is used with integrated wireless communications systems due to its ability to connect to Wi-Fi networks to act as an access point, allowing other devices to connect to it and exchange data. It can be linked with Li-Fi technology. By connecting the ESP8266, a local Wi-Fi network is created and controlled by programming it. In this case, it works as a controller to send data via visible light by connecting to the building’s lighting system. Data can be sent within the entire building; while on the receiving side, an Arduino Uno controller was used. Here, communication is in one direction and can be implemented via more than one MIMO receiver. Figure 9 shows the practical implementation of integration between the two technologies.

C. BPL system design

**Figure 10:**
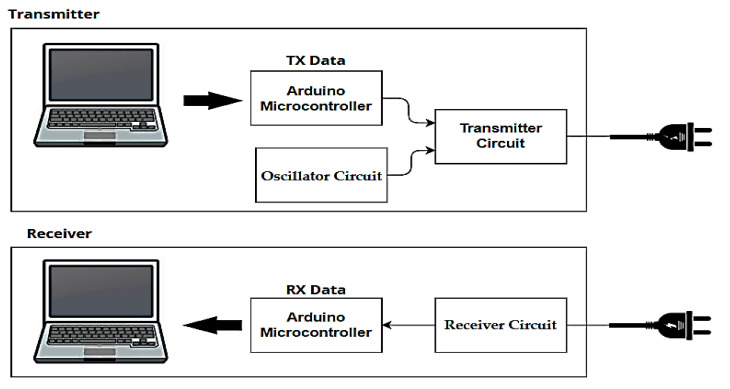
Proposed block diagram of the BPL system.

As a result of the difference in quality and performance of electrical devices and equipment, they may cause interference, noise, and harmonics through the power lines connected to them, and therefore when designing a BPL system, it is necessary to consider increasing the quality of signal transmission. Figure 10 shows the block diagram of the proposed BPL system design. In this design, it is proposed to effectively filter the data signal from any noise, and the BPL system can be designed according to the data flow method to transmit or receive data using an asynchronous serial communication system. It is characterized by being simple, low cost, and applicable in any environment with a high reliability in sending and receiving data. 

**Figure 11:**
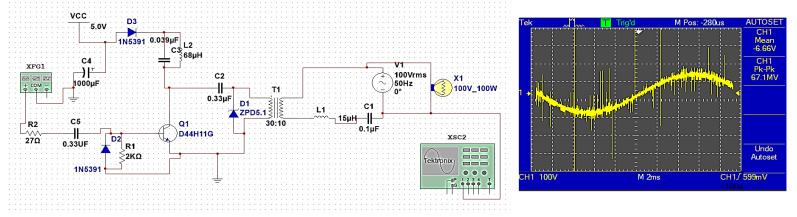
BPL transmission circuit and the carrier module output signal.

Data are sent through the sending computer to the Arduino Uno controller and then to the transmission circuit shown in Figure 11. But for it to pass through the low-voltage power line of 220 V, 50 Hz without any harmonics or effects on the signals, it needs modification.

**Figure 12:**
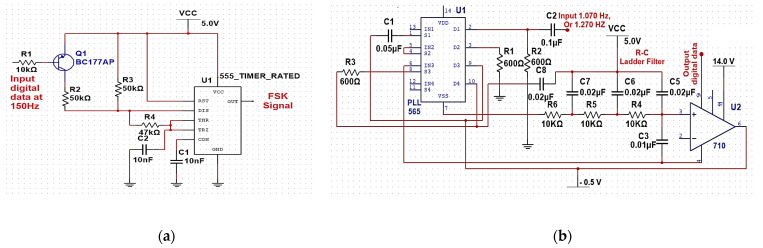
Circuit diagram of (**a**) the FSK modulator and (**b**) the FSK demodulator.

FSK modulation is shown in Figure 12a, which is considered a category of single-carrier modulation. It was proposed to modify the data signal, using a simple oscillator circuit using IC555, to generate the carrier wave to obtain the modified wave [20]. Then, the data were converted from the form of digital logic (square signal) into a sine wave capable of passing through the transmitter circuit, and this is performed by the resonance circuit in the transmitter circuit, and from there to the electrical socket to be sent through the power line as shown in the proposed block diagram shown in Figure 10.

The transmission circuit shown in Figure 11 consists of a resonance circuit consisting of L2 and C3, which works to convert the square wave issued by the FSK rectifier into a sine wave, and then to amplify the signal via Q1. C5 and R2 are used to protect the circuit from excessive currents. The transformer and a low-pass filter circuit consisting of L1 and C1 isolate the electricity signal from the data signal to be transmitted.

The transmitter circuit is like an inverter circuit. It converts the signal issued from the 220 V power line with a frequency of 50 Hz into a signal carrying the same voltage of 220 V but with very high frequencies (the modified signal), and then it is connected to a low-pass filter circuit consisting of a coil and a capacitor, to prevent low frequencies represented by a power line signal with a frequency of 50 Hz. If there is no data to be sent, the FSK unit is on the transmitting side, and the signal is in the form of a straight line, while if there is data to be sent, the signal at the transmitting end is a square wave signal.

The presence of an oscillator in the BPL technology can generate certain frequencies through the instructions issued by the Arduino, depending on the data to be sent, represented by the frequency of the carrier wave, and thus helping to convert the data into an electrical signal that can be transmitted and modified, which improves the performance and efficiency of the BPL system and allows data to be transmitted via the electrical power distribution network.

**Figure 13:**
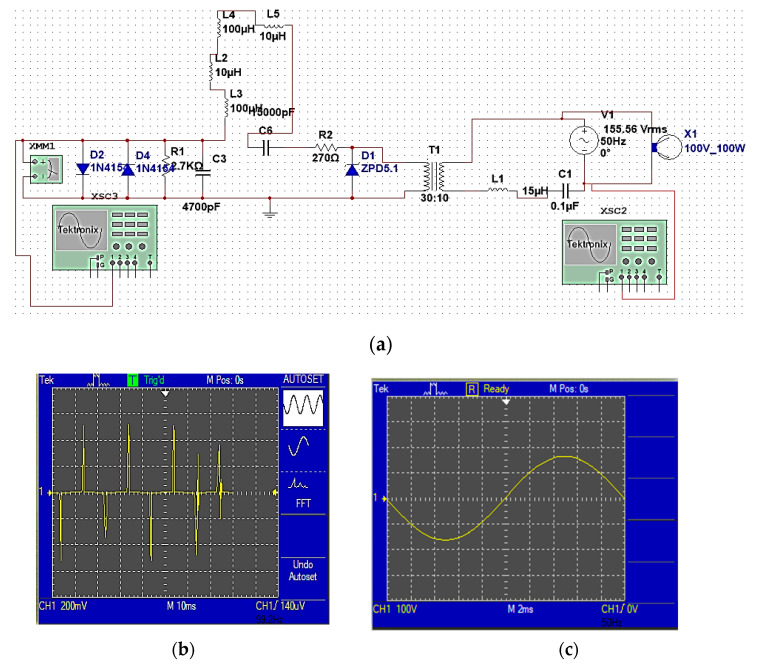
(**a**) The BPL receiver circuit, (**b**) the actual extracted and received data signal, and (**c**) the electrical supply line signal.

The receiver circuit is shown in Figure 13. It can work as if it were a data detection circuit without affecting the electrical signals that pass through the wires that supply power to the building or on the device connected to the electrical socket.

On the receiving side, the transmitted data signal is received through the electrical socket connected to it. The electrical power line works as if it were a wired communication channel. Then, the signal is transmitted to the receiving circuit to receive the data-modulated sine wave signal. It consists of an isolation circuit containing the transformer and a resonance circuit consisting of L1 and C1, which works to isolate the electrical signal from the data signal to be received and can produce the specific resonance frequency represented by the data signal. The resonance circuit selects the frequency as it happens with a radio device, while the coils L2, L3, L4, and L5 and the capacitor C3 act as a filter to purify the signal and process the waveform. A stable sinusoidal signal is obtained as shown in Figure 13c. Diodes D2 and D4 work to separate the signals and obtain high-frequency data as shown in Figure 13b, and also to control the flow of current through the circuit and improve the performance of the circuit. Through the FSK modification detection circuit shown in Figure 12b [20], the data signal is reshaped in its natural form so that the output of the receiver circuit is connected to the Arduino controller to be displayed on the receiver’s computer. Figure 13b,c show the extracted data signal and the power line signal.

A zero-crossover detection circuit can be used on the receiving side to identify the presence of any data across the feed line when the sine wave passes through the zero point. It is a small circuit based on an optocoupler. Despite the simplicity of the circuit, it is used to control the timing of the encoding and decoding process of data transmitted through electrical power lines. It maintains the synchronization of the process of receiving data via BPL. This synchronization contributes to improving the performance of data transfer and reducing interference resulting from changes in the voltage of the supply line [21], thus the possibility of receiving data in its natural form without any change in its capacity or form. 

**Figure 14:**
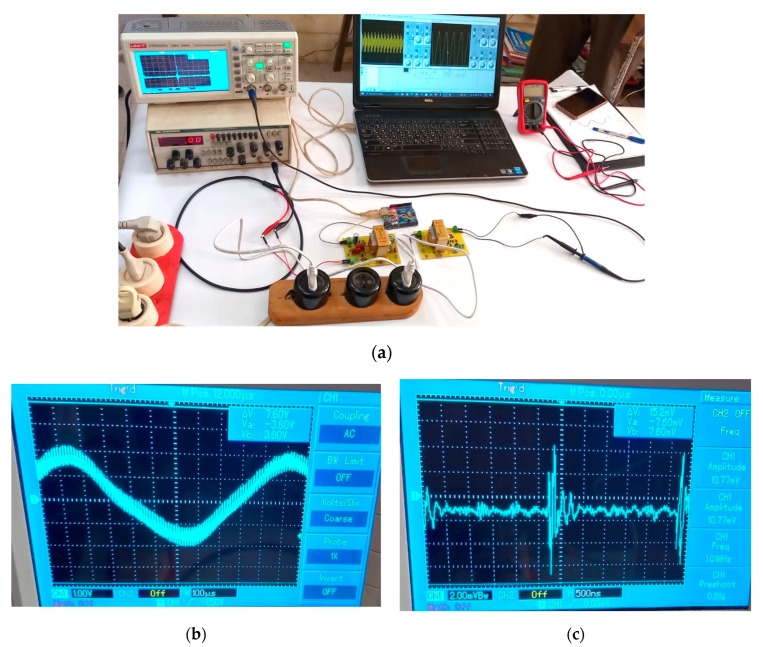
(**a**) The practical implementation of the BPL system, (**b**) the modified data signal, and (**c**) the actual extracted and received data signal.

Through simulation, it was found that the BPL system can send data with a bandwidth of up to 500 KHz as the carrier frequency of the data signal (modulated wave) across a low-voltage power line. Therefore, the high-frequency signal can be sent on the low-voltage power line. While implemented practically, as shown in Figure 14, its bandwidth reaches 200 KHz, and this is using an oscillator generator with a voltage of 220 V and in the sine wave mode and connecting it to the transmitter circuit, which is also connected to the Arduino controller. The system works stably because the actual signal values resulting from the simulation are close to theoretical values. The implementation was carried out in one phase, but this can be successfully achieved across three phases of the electrical power line.

D. Integration between BPL and Wi-Fi

This is known as a hybrid BPL (PLC)/Wi-Fi network, which represents an innovative solution for providing a reliable and high-speed internet connection. This combination aims to take advantage of the advantages of each technology to meet the needs of modern networks. BPL uses power lines to transmit data, while Wi-Fi provides wireless connectivity to devices. It has recently spread in places where there is no wireless coverage or weak signals are present, especially in remote or rural areas, and its spread is expected to increase in the future [22]. Good infrastructure and power lines can provide high data transmission efficiency over BPL, which may reach Gbps, and thus will be an alternative to designing the infrastructure needed to provide a digital subscriber line (DSL), which operates at speeds ranging from 500 Kbps to 3 Mbps [4]. There are also several protocols that can be used to achieve network security mechanisms, such as the EAP protocol, which is a flexible protocol based on a framework to protect wireless and wired networks by controlling authentication [22].

**Figure 15:**
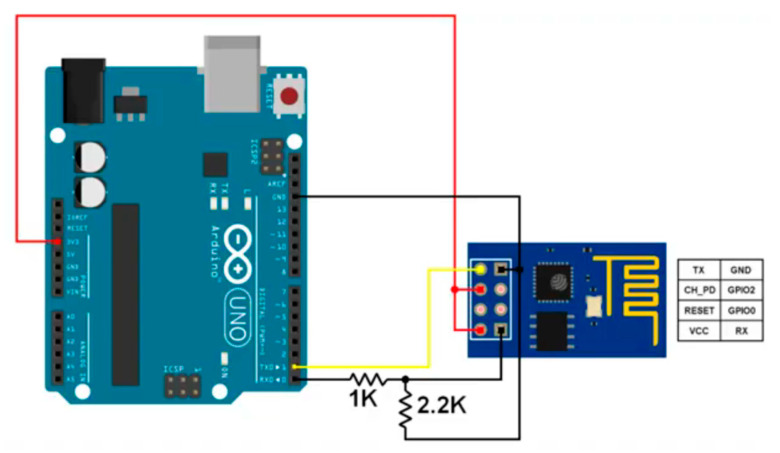
Proposed connection for the integration between BPL technology and Wi-Fi technology.

In the context of BPL and Wi-Fi integration, ESP8266, an integrated circuit with a microcontroller, was used, as explained earlier [23]. ESP8266 provides the ability to connect to Wi-Fi networks, it is programmed as a small Wi-Fi module to connect to the local Wi-Fi network and send and receive data serially to work within the BPL technology system, and it is connected as shown in Figure 15. ESP8266 operates at 3.3 V, so it uses two resistors, one 1 KΩ and one 2.2 KΩ, to reduce the voltage coming from the Arduino 5 V to 3.3 V to maintain the data transfer process. ESP8266 works as part of a router system that uses BPL technology to connect devices to the internet via power lines. By configuring and programming it, IP addresses are obtained, which enables it to act as a server and the devices connected to the network to act as its clients. ESP8266 can also connect and control other devices to the local wireless network, such as IoT applications; direct data traffic via BPL, which enhances connectivity in smart environments; and wireless sensor network applications to collect data from sensors (such as temperature, humidity, or traffic) and send it to a server or cloud platform. This integration is achieved by using ESP8266 to ensure data load balancing and improve network performance.

E. Integration between BPL and Li-Fi

BPL is an innovative solution for improving connectivity and data transmission in different environments. By leveraging the advantages of each technology, BPL can be used to extend coverage and provide connectivity in areas where Wi-Fi or fiber optics are difficult to use or where traditional internet infrastructure services are not available, while Li-Fi provides fast and secure connectivity in environments that require direct line of sight, supporting smart applications and improving overall network efficiency.

Although BPL and Li-Fi are two different technologies, as shown in Table 1, they can be combined to improve wired and wireless communications, keeping information confidentiality higher and taking advantage of the benefits of Li-Fi technology.

Achieving integration is possible after transmitting data over the power line through the proposed BPL transmission circuit in Figure 11, which is connected via SSR to control the intensity of the LED lamp to obtain OOK modulation. The electrical signal (data) is converted into light and received through the proposed Li-Fi receiving circuit in Figure 5, which includes a photodiode to detect the converted light and convert it back into an electrical signal. Due to the devices connected to the power line, interference may appear between the electrical signals generated by the devices and the transmitted and received signals, which may lead to data loss or signal-quality degradation. To reduce the interference, filters are required to filter signals or isolate circuits.

A photocoupler can also be used to act as a link between BPL and Li-Fi. The photocoupler is connected to the proposed BPL transmission circuit in Figure 11, which is connected to the power line to transmit data [24]. Isolation and signal processing will be achieved. In this case, the photocoupler converts electrical signals transmitted via power lines into light signals that can be received via Li-Fi without loss of quality. The electrical signal to be converted is connected via an LED circuit inside the photocoupler and produces light. The intensity of the resulting light is proportional to the level of the input electrical signal.

The light emission from the LED can be utilized and sensed by the phototransistor, which is the internal component of the photocoupler, to detect the light and convert it back into an electrical signal, which can be received by the BPL receiving circuit as explained in Section 4.C, or received by exchanging data over a LAN designed by ESP8266 as explained in Section 4.D.

The photocoupler achieves electrical isolation between the transmitter and receiver, as well as between devices connected via power lines, protecting against interference, noise, and data breaches. It can also transmit data over power lines with high response speed and accuracy, thus improving network performance and expanding the communication range.

## 5. Detection and Stopping Threats in the Integration of BPL and Li-Fi

BPL can be compromised by attackers sending interference signals on the same frequencies used in BPL, or accessing the electrical wires used in the BPL network and intercepting the transmitted signals. This can lead to eavesdropping on the network, tampering with and losing data, spreading malware on devices connected to the network, or stopping the connection. Despite the advantages of BPL, it is considered a threat in security, military, and nuclear energy networks. Similarly, despite the advantages of Li-Fi, it is not completely immune to hacking. It can be hacked by using unsecured LED lights, and thus the possibility of capturing light signals using special equipment by attackers and thus obtaining unencrypted signals or causing interference with the original signals, leading to data loss or hacking.

A. Threat detection

The network should be constantly monitored to detect any hacking attempts or interference. Intrusions can be detected when Li-Fi and BPL are integrated, by using optical sensors such as phototransistors or CMOS image sensors to detect Li-Fi signals. They receive Li-Fi signals and when the light intensity from a Li-Fi source (such as an LED) is modified, the sensor captures these changes and converts them into an electrical current and then to the Arduino, to analyze the data to monitor unusual changes and interferences in the light intensity, which may indicate hacking attempts.

**Figure 16:**
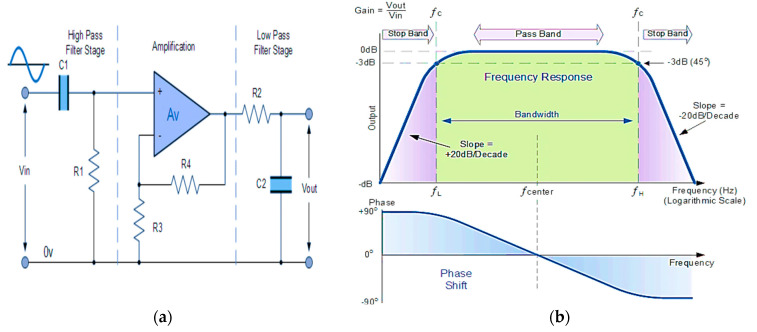
Active broadband filter, (**a**) circuit diagram, and (**b**) the frequency response curve.

Similarly, Active Broadband Filters (ABPFs) with an Optical Spectrum Analyzer (OSE) can be used to analyze the light signals by measuring the optical spectrum of different radiation and identify any unwanted interference. An ABPF is a widely used filter in electronic systems. It allows a specific frequency range to pass while stopping frequencies lower and higher than this range [25]. As shown in Figure 16, through the Arduino connected to both ABPFs and the OSE, the signals are analyzed and frequencies outside this range are blocked. ABPFs can be integrated with other security systems such as Intrusion Detection Systems (IDSs) to enhance protection against threats. It is the best option as it helps with early detection of potential threats by monitoring the frequencies used in BPL and Li-Fi, improves the quality of the received signal, and helps in eliminating noise and reducing errors and interference between signals.

B. Stopping Threats

**Figure 17:**
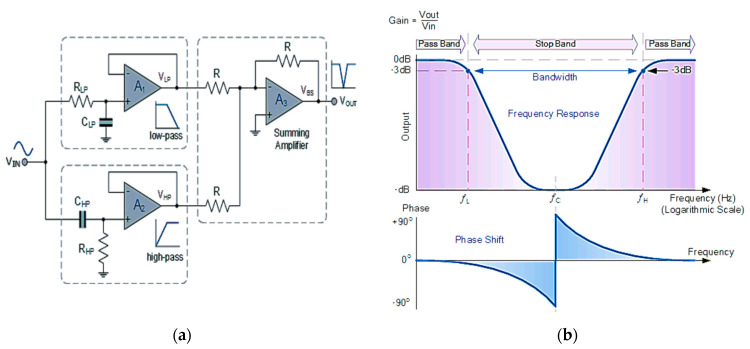
Active stop band filter, (**a**) circuit diagram, and (**b**) the frequency response curve.

The active stop band filter is an effective tool in enhancing network security. It works in the opposite way to the pass band filter [25]. As shown in Figure 17. It filters or stops a specific range of frequencies associated with threats and unwanted interferences that are identified in advance, so that data can be transmitted properly. The Arduino is programmed to select only the desired frequencies and then they are passed through the filter. It helps to eliminate noise, improve signal quality, reduce the chances of hacking, and enhance security.

## 6. Application

The integration of Wi-Fi, Li-Fi, and BPL technologies offers promising potential for developing secure and efficient communications systems. This integration can enhance the integrated internal network, improve security and privacy, and increase operational efficiency while reducing costs. The proposed integrated technology can be applied as follows:

A. Smart homes and cities

Smart grid management and environmental sensing can be improved with appropriate sensors, enhancing operational efficiency and rapid response in emergencies. Wi-Fi and Li-Fi integration can be used to provide high-speed public communications in open spaces [7]. Li-Fi can be used to provide secure and fast communications in sensitive areas such as meeting rooms, private offices, or inside homes [23]. BPL can be used to transmit data across the power grid inside a home and neighboring homes, especially in hard-to-reach areas. To implement this, unified management systems must be implemented to coordinate the work of these technologies and make the best out of them [16].

B. Hospitals and health centers

The integration of the proposed technologies provides fast and reliable communication for transferring medical data between medical devices and electronic patient files, as well as communication and collaboration between doctors and patients, thus enabling the application of remote healthcare and continuous monitoring of patients [8]. Li-Fi can be used alone to provide secure communications in operating rooms and sensitive areas, ensuring that signals do not interfere with medical devices [12]. The integration of Wi-Fi and Li-Fi can also be used to provide general communications for staff and visitors. The integration of BPL and Li-Fi can be used to connect medical devices and systems to a reliable internal network, achieving advanced security measures to protect sensitive data.

C. Education and research organizations

The integration of the proposed technologies can provide an advanced learning environment with distance learning capabilities, quick access to digital resources, and improved interaction and collaboration between students and teachers. Li-Fi technology can be used alone in classrooms and laboratories to provide secure and fast communications, which can be used as a live broadcast to students or staff of the educational institution or as a warning tool. The integration of Wi-Fi and Li-Fi can also be used in common spaces and open areas. In addition, the integration of BPL and Wi-Fi can be used to connect different buildings and departments to a unified internal network.

D. Industry

The integration of Wi-Fi, Li-Fi, and BPL technologies can enhance automation and digitization in production lines through fast communication and data reliability. It also helps improve maintenance efficiency and rapid response to operational problems by implementing sensor networks that operate through these technologies. Li-Fi can be used in production lines and sensitive areas, providing secure and reliable communication without interference with industrial devices [26]. The integration of Wi-Fi and BPL can also be used to provide integrated services to workers and visitors.

E. Transportation

The integration of the proposed technologies can be used to improve communications and monitoring in transportation networks such as railways, roads, and ports [8]. Li-Fi can be employed to provide secure and fast communication between vehicles for smart transportation applications and indoor infrastructure systems for airports and stations [27]. The integration of Wi-Fi and Li-Fi can also be used to enable wireless communication for passengers on board transportation or between transportation systems and vehicles to monitor traffic and congestion on roads. In addition, the integration of BPL and Li-Fi can be used to transfer operation and maintenance data, as well as data provided to workers or passengers, in an efficient manner.

F. Propaganda and advertising

This integration of the proposed technologies can be used to create advanced and highly efficient digital advertising and guidance environment in tourist, historical, and religious places and organizational places for celebrations and sports such as organizing the World Cup, archaeological museums, Hajj season organizations, and others. Using Li-Fi, it is possible to create digital billboards that interact with users as they approach them, displaying personalized and interactive content for augmented reality and virtual reality applications. BPL can also be used to link billboards together to update content and data quickly and efficiently, and Wi-Fi can be used to connect mobile devices to the advertising network.

G. The military field

The integration of Wi-Fi, Li-Fi, and BPL technologies will contribute to enhancing military capabilities in the areas of communications, reconnaissance, logistics support, and training. The integration of Wi-Fi and Li-Fi can be used to provide secure wireless communication between forces and military centers in different regions. Li-Fi can be used to transmit confidential and sensitive data at high speed and with high security [12]. In addition, the integration of BPL and Li-Fi can be used to support military communications infrastructure, supply management, and link monitoring and sensing systems, whether in field sites or in remote areas.

## 7. Conclusions

In the rapidly evolving world of communication, there is an urgent need to develop technologies to meet the increasing requirements of users. In this context, this paper presents three technologies that have become among the most important technologies used at the present time. They are Li-Fi, Wi-Fi, and BPL technologies to work individually or to integrate with each other, to benefit from the advantages of each technology, and to achieve a qualitative shift in the field of wired and wireless communications. Li-Fi technology works on transmitting and receiving via light and offers high data transfer capacities and high security compared to traditional technologies. At the same time, Wi-Fi is characterized by its ubiquity and ability to provide fast and flexible connectivity. On the other hand, BPL plays an important role in providing internet services over electricity infrastructure, providing universal coverage and low cost. 

In this paper, simple and low-cost circuits are proposed to implement each technology to take advantage of the different advantages offered by each technology. The ESP8266 module is proposed to integrate Li-Fi and Wi-Fi, and integrate BPL and Wi-Fi, which is characterized by low power consumption, easy programming, availability, and low cost. A photocoupler is also proposed to integrate BPL and Li-Fi, which is characterized by its availability, low cost, high response speed, and accuracy in optical data transmission. This integration of technologies will improve efficiency and reliability, increase data speeds, reduce costs, utilize infrastructure, expand coverage, and meet user needs in different scenarios and applications. This paper also mentions some of the applications that can be implemented that support this integration. Furthermore, OSE and filters are effectively used to prevent attacks that may occur in sensitive and military networks.

## Figures and Tables

**Table 1:** Comparison between Li-Fi technology, Wi-Fi technology and BPL technology.

Feature	Li-Fi	Wi-Fi	BPL
Full form	Light fidelity	Wireless fidelity	Broadband over power lines
Based on	Radio signal waves (EM waves “RF”).	Light signals (visible light “VL”).	It relies on transmitting data via an electrical power line.
Operation	Li-Fi transmits data using light with the help of LED bulbs.	Wi-Fi transmits data using radio waves with the help of a Wi-Fi router.	BPL uses electrical power infrastructure to transmit data.
Interference	Does not have any interference issues like radio frequency waves.	Will have interference issues from nearby access points (routers).	It may experience interference with other devices connected to the same power infrastructure.
Technology	Presents IrDA-compliant devices.	WLAN 802.11a/b/g/n/ac/ad standard compliant devices.	It relies on digital packet technologies, UPA, and IEEE P190.
Applications	Used in airlines, undersea explorations, operation theaters in the hospitals, and office and home premises for data transfer and internet browsing.	Used for internet browsing with the help of Wi-Fi hotspots.	Suitable for applications requiring wide coverage such as public utility networks.
Advantages	Interference is less, can pass through salty sea water, works in dense regions.	Interference is more, cannot pass through sea water, works in less dense regions.	Uses existing electrical power infrastructure and has wide geographical coverage.
Privacy	In Li-Fi, light is blocked by the walls and hence will provide more secure data transfer.	In Wi-Fi, RF signals cannot be blocked by the walls and hence needs to employ techniques to achieve secure data transfer.	Takes advantage of existing electricity infrastructure, so may be more vulnerable to hacking.
Data transfer speed	Up to speeds of up to 224 Gbps.	Offers speeds of up to 9.6 Gbps in modern standards.	Speeds ranging from 2 to 200 Mbps.
Frequency of operation	Uses visible and infrared light frequencies.	Operates in the 2.4 GHz and 5 GHz radio frequency bands.	Uses the frequency of electrical power lines (50–60 Hz).
Data density	Works in a highly dense environment due to its optical bandwidth.	Works in less dense environments due to interference-related issues	It is limited due to technical limitations of data transmission over power lines.
Coverage distance	Limited by line of sight and often no more than 10 m.	About 32 m (WLAN 802.11b/11g), varies based on transmit power and antenna type.	Covers a wide geographical area up to more than 10 m.
System components	Uses an optical transmitter and a receiver.	Requires a wireless access point and multiple compatible devices.	Uses existing electrical power infrastructure and devices connected to the grid.

## Data Availability

All data are included in the article.
